# Compromising improves forecasting

**DOI:** 10.1098/rsos.221216

**Published:** 2023-05-17

**Authors:** Dardo N. Ferreiro, Ophelia Deroy, Bahador Bahrami

**Affiliations:** ^1^ Faculty of General Psychology and Education, Ludwig Maximilian University, Munich, Germany; ^2^ Munich Center for Neuroscience, Ludwig Maximilian University, Munich, Germany; ^3^ Faculty of Philosophy and Philosophy and Science, Ludwig Maximilian University, Munich, Germany; ^4^ Division of Neurobiology, Faculty of Biology, Ludwig Maximilian University, Planegg-Martinsried, Germany; ^5^ Institute of Philosophy, School of Advanced Study, University of London, London, UK; ^6^ Centre for Adaptive Rationality, Max Planck Institute for Human Development, Berlin, Germany

**Keywords:** forecasting accuracy, collective decisions, wisdom of crowds, group decision-making

## Abstract

Predicting the future can bring enormous advantages. Across the ages, reliance on supernatural foreseeing was substituted by the opinion of expert forecasters, and now by collective intelligence approaches which draw on many non-expert forecasters. Yet all of these approaches continue to see individual forecasts as the key unit on which accuracy is determined. Here, we hypothesize that compromise forecasts, defined as the average prediction in a group, represent a better way to harness collective predictive intelligence. We test this by analysing 5 years of data from the Good Judgement Project and comparing the accuracy of individual versus compromise forecasts. Furthermore, given that an accurate forecast is only useful if timely, we analyze how the accuracy changes through time as the events approach. We found that compromise forecasts are more accurate, and that this advantage persists through time, though accuracy varies. Contrary to what was expected (i.e. a monotonous increase in forecasting accuracy as time passes), forecasting error for individuals and for team compromise starts its decline around two months prior to the event. Overall, we offer a method of aggregating forecasts to improve accuracy, which can be straightforwardly applied in noisy real-world settings.

## 1. Introduction

Cresus, who ruled over Lydia in the 6th century BC, tested the oracles of his world to discover which gave the most accurate prophecies. He sent out emissaries to seven oracles and asked what the king would be doing exactly 100 days after the emissaries' departure from the palace. The king declared the oracle at Delphi the most accurate, as she rightly foresaw that the king would be making a lamb-and-tortoise stew on that very day.

Oracles have been replaced by probabilistic forecasts (following [1]) but Cresus’s comparative approach has continued to be overshadowed by unquestioned trust in expert forecasters (see [2]). Cresus's approach was resurrected in 2011 when researchers from five universities competed to find out who the best forecasters would be. They joined a forecasting tournament organized by the Intelligence Advanced Research Projects Activity (IARPA) in the United States, entering the first 2 years of the tournament with about 1600 pre-selected participants, who went on to be assigned to various forms of training, teaming and tracking. Beating Cresus’s test in terms of systematicity, the enrolled forecasters were asked to provide and update their probabilistic forecasts on at least 25 out of hundreds of standing geopolitical issues, with many responding to more.

The winner of the first two rounds of the competition, which would later be known as the Good Judgement Project, combined a set of training and skill tracking which could produce better forecasts [3]. The most surprising finding however came as a direct challenge to individualistic models of forecasting that have persisted so far in most governmental agencies, and continue to govern for instance prediction markets [4] and expert judgement aggregation techniques (e.g. [5]). Against the warnings of group-think and herding [6,7], individuals who had direct and continuous discussions with each other gave more accurate predictions than isolated individuals. They also were better than individuals who could gather social information but not discuss. As an additional confirmation, individual characteristics facilitating deliberation with others, such as cognitive flexibility and open-mindedness [8], cooperativeness within teams [9] as well as the tendency to provide more articulate rationales [10] could distinguish the best forecasters in the competition.

The Good Judgement Project has now gone through many more rounds of iterations and has, in the process, become a brand and a household name for forecasting. It has also made a significant contribution to the field by making a large bulk of forecasting data available for others to use for their own research. We were interested in pushing the question further and ask whether one could go beyond identifying the best forecasters, and find new ways to make the best of these forecasts.

Here we were particularly interested in comparing the benefits of combining individual forecasts with those of combining compromises between individuals. In other words, instead of considering the respective accuracy of each and every individual prediction, we reconstructed what would happen in a parliament of forecasters, where groups would be assigned a compromise by reconciling their members' various predictions through a fair averaging method. The reason to equate simple averaging with compromise comes from the contrast with a consensus that would rest on a negotiated convergence. Averaging is a common method for calculating hypothetical collective decisions (e.g. statisticized groups) which has the benefit of weighing every opinion equally. By contrast, weighing opinions by some predefined measure of competence (e.g. [11]) introduces inequality by definition which goes against the democratic potential of forecasting tournaments (cf. [12] and discussed in [13]). Weighing by competence privileges people who were right in the past. Though weighing by competence could eventually be sound, and averaging is not always explicitly recognized as a good rule of decision [14], it remains true that descriptively, many actual collective decisions do indeed approximate a simple averaging rule ([15], see also [16] for a historical approach) notably in forecasting [13].

The mechanisms behind the benefits of compromise come from combining the benefits of pooling across the diversity of packets of information partially available to group members ([17,18], see also [19]) together with deliberation and exchange of arguments [20]. Recently, Satopää *et al*. [21] provided a useful approximation for the source of collective benefits in forecasting that may result from social interaction between forecasters. They concluded that, when competitive forecasters are allowed to discuss their predictions with one another, about 25% of the accuracy improvement comes from information improvement. Those who possess some useful information tell others about it and everyone is better off. Another 25% of the forecasting improvement, Satopää *et al*. attributed to bias reduction. Bias reduction through interaction may come from receiving cautionary advice from team mates about important issues such as respecting base rates and avoiding representativeness bias. However, the major effect of collective interaction, accounting for 50% of the benefits, comes from noise reduction. When many independently generated opinions are aggregated, uncorrelated noise in various opinions should cancel each other out (smaller benefits are expected from calibration of confidence [22] but see [23] for moderate optimism within superforecasters). While the impact of social interaction information and bias are adequately achieved in small-scale interactive groups [24], the well-documented benefits of averaging on noise reduction are believed to be more pronounced with larger groups, as also reported in classic ‘wisdom of the crowds’ [25,26] and formalized in Condorcet's Jury Theorem.

Here, we report results of the comparison between two different ways of calculating how far off or close forecasters were in their predictions: individual responses after discussion, and local compromises in groups. In addition, we also tested Navajas *et al*. [27]'s model of second-order compromise, which averages across compromises within groups. In their general knowledge survey, averaging consensual answers reached by small groups leads to more accurate answers than averaging individual answers, yet the best performance in their experiment was achieved by averaging consensual answers across different groups, local and global.

Determining the better forecasts should account not only for accuracy but also for timing. As announced on the website of the Good Judgement Project, the goal is to ‘see the future sooner’. Rightly predicting that the price of oil will be 200USD next January for instance is good, but predicting it in September is better than predicting it in December. If forecasts are meant to help with planning, having the most accurate forecasts earlier also matters.

Therefore we were interested in finding out whether compromise forecasts would be better not only in their end results but also how they compared through time with the aggregate of individual forecasts. To do so we consider the prediction errors ([28]; known as ‘Brier scores’ or ‘forecasting error’, based on the difference between a probabilistic forecast and the actual truth value of the final report of the event) provided by individuals from interactive groups of the Good Judgement Project, who registered forecasts between 2011 and 2015.

Our results show that harnessing the benefits of deliberation is better served by compromise forecasts than individual forecasts: averaged predictions were consistently more accurate through time.

## 2. Methods

We report a reanalysis of the data reported originally by the Good Judgement Project [29] (‘GJP Data’, https://doi.org/10.7910/DVN/BPCDH5, Harvard Dataverse, V1; https://dataverse.harvard.edu/dataverse/gjp). These data (and the details of the experimental procedures from which they were obtained) have already been described in a number of previous publications (e.g. [8,9,30,31]).

### 2.1. Source

Between 2011 and 2015, the Good Judgement Project recruited people online to participate in a forecasting tournament funded by the Intelligence Advanced Research Project Activity (IARPA, U.S. government) aiming to produce accurate geopolitical forecasts. Forecasting questions, called Individual Forecasting Problems (IFPs), were released periodically by the IARPA. Participants posted their forecasts up until a designated deadline indicated in each IFP. The team with the best forecasting accuracy would win the competition. Some examples of IFPs raised in 2011 for instance would include:

— ‘Will Joseph Kabila remain president of the Democratic Republic of the Congo through 31 January 2012?’
— ‘Will a foreign or multinational military force fire on, invade, or enter Iran before 1 September 2012?’

For a complete list of questions see the file ‘ifps’ in the ‘GJP Data’ as described above.

Within the Good Judgement Project website, recruited participants could log on as often as they wanted and post a new forecast (or update an existing one) on all open IFPs. For binary questions, such as the examples above, forecasts consisted of a probability estimate in the form of a number ranging from 0 (absolutely not) to 100 (absolutely yes). The source dataset consists of the IFPs, the forecast estimates of each anonymised participant and the eventual fate of each IFP as transpired by the corresponding deadline. The dataset also includes extensive demographic information about the participants.

## 3. Data analysis

### 3.1. Preprocessing

In this study, we focus on the forecasting performance through time, with different means of aggregation, as a function of the time between the submitted forecasts and the respective question deadline. From the original source data, we only analysed responses which obeyed the following inclusion criteria:

— Responses to binary (yes or no, as possible outcomes) questions.
— Members of interactive teams.
— For each time interval analysed, a minimum of 5 forecasts per team were required.

We decided to exclude two teams which we deemed outliers (Teams 1 and 2 from the original data structure). The reason being that these teams had 2157 and 273 participants, which was deemed detrimental to genuine interaction (see electronic supplementary material, figure S1 for Team sizes)

The resulting dataset spans 88 teams (7 ≤ team size ≤ 78), totaling 3067 individual forecasters. Individuals registered forecasts in response to 382 questions administered between 2011 and 2015. Participants had been selected to enter the project and were therefore not representative of the general public: they were predominantly males (81%) and US citizens (71%); their average age was 39 ± 14 (mean ± s.d.) and their level of education high (77% held a bachelor's degree, 50% a master/professional degree and 11% a doctorate degree). In addition, across the different years of the projects, individual forecasters went through different kinds of training.

### 3.2. Quantifying forecast error

Our focus was the binary IFPs, i.e. Individual Forecasting Problems where users had to assign a probability to whether a given event would happen in the future. Because all questions in the database had already been concluded, the correct outcome for each IFPs was known to us. Therefore, for each forecast entered by each participant at any point we could calculate a forecast error score. We used the Brier scoring rule [28]. Brier scores are the sum of the squared deviation between the forecasted probability and the real outcome:

3.1 $$\mathrm{Brier}\;\mathrm{Score}={\left({\mathrm{Forecast}}_{\mathrm{yes}}-\mathrm{Outcome}\right)}^{2}+{\left({\mathrm{Forecast}}_{\mathrm{no}}-(1-\mathrm{Outcome})\right)}^{2},$$

where Forecast_yes_ and Forecast_no_ are the complementary probabilities assigned to the event happening and not happening, respectively. *Outcome* is 1 if the event did happen and 0 otherwise. For example, if I assign a 0.7 probability to raining tomorrow, and it does end up raining, the Brier score of my forecast would be calculated as follows:

$$\mathrm{Brier}\;\mathrm{Score}={\left(0.7-1\right)}^{2}+(0.3-0)2=0.18.$$

Possible scores range from 0 indicating perfect accuracy, e.g. forecasting rain with certain (100%) probability and having rain the next day, to 2 indicating a hopelessly wrong opinion, e.g. forecasting rain with 100% probability followed by a dry day. A chance level (50–50) forecast would always return a Brier score of 0.5. Because higher Brier scores indicate lower prediction accuracy we refer to Brier scores as a measurement of ‘forecasting error’ interchangeably.

### 3.3. Aggregation of forecasts

**Figure 1.**
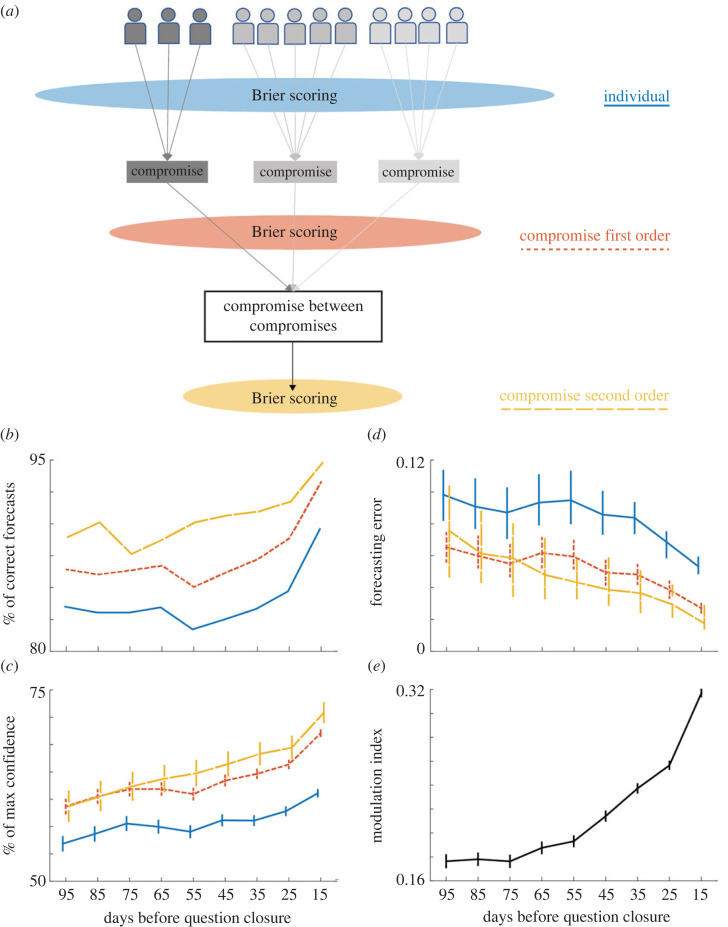
(*a*) Schematic representation of the different methods of aggregation of opinions. Individuals who interacted in the same group appear in the same shade of gray. Coloured ellipses depict the three different opinion aggregations (i.e. at which point of aggregation the Brier scoring took place). Text legends to the right include the line type that represents each aggregation on panels B-D (Individual, 1st order and 2nd order Compromise data points are represented by blue solid lines, red short-dash lines and yellow long-dash lines respectively). (*b*–*e*) Forecasting through time. Time axis depicts the middle point of the corresponding 30 day time bin. (*b*) Percentage of correct forecasts. (*c*) Percentage of maximum possible confidence (mean ± standard error). (*d*) Median and 95% confidence interval of forecasting error (Brier score). Blue solid: Individual Brier scores. Red short-dash: 1st order aggregation, i.e. within team compromise forecast (average forecasts within team members before Brier score calculation). Yellow long-dash: 2nd order aggregation, i.e. across team compromise forecast (average forecasts within team members first, then average compromise forecasts across teams). Lines in panels C and D have been shifted slightly in the time axis only for visualization purposes. (*e*) Modulation index comparing the individual (D, blue solid line) and the 1st order compromise (D, red short-dash line) forecasting error (mean ± standard error).

In the Good Judgement Project [29], ‘GJP Data’, https://doi.org/10.7910/DVN/BPCDH5, Harvard Dataverse, V1; https://dataverse.harvard.edu/dataverse/gjp), participants were assigned to teams, who communicated via an online forum which allowed them to engage in discussions. Team members entered their forecasts individually. All forecasts submitted during the time window and/or team being analysed were included in our calculations. We used these individual forecasts to calculate group performance in terms of Brier Score. Thus we could compare forecast accuracy in three different ways (figure 1*a*). First, by calculating the Brier score of each individual probabilistic forecast, and then averaging the scores across team members we obtained the average ‘Individual Forecasting Error’. This gives us a measure of the average quality of the individual members of different teams. Second, by averaging the probability forecasts cast by the team members, we calculated a within-team compromise forecast (as well as by reshuffling across-teams, see electronic supplementary material, figure S3). The Brier score of this compromise was defined as the ‘1st order Compromise Forecasting Error’.

Finally, by averaging the 1st order compromise probability forecasts, we calculated a higher order global compromise forecast across teams. The Brier score of such ‘compromise between compromises', was defined as the ‘2nd order Compromise Forecasting Error’. These measures match the wisdom of structured crowds as originally proposed by Navajas *et al*. [27].

### 3.4. Forecast confidence

The confidence of each forecast probability estimate was defined as the absolute difference between the assigned probability to the forecast and chance level (i.e. 0.5). Therefore, forecasts of 0.7 and 0.3 would predict the opposite outcome for a given IFP but with equal confidence of 0.2.

### 3.5. Modulation index

To compare the relative performance of the individuals and the 1st order compromise forecasting, we calculated a modulation index (MI) as follows:

3.2 $$\mathrm{MI}=\frac{\mathrm{Individual}-\mathrm{Compromise}}{\mathrm{Individual}+\mathrm{Compromise}},$$

where *Individual* is the average Brier score of individual probability estimates across team members, and *Compromise* is the Brier score of the average probability estimate across team members (i.e. 1st order compromise; figure 1*e*).

### 3.6. Statistics

A generalized linear mixed-effects model (GLM) was calculated to test whether there was a time and/or an aggregation level effect in the forecasting error, using the matlab function ‘fitglme’ according to the linear model:

3.3 $${\mathrm{Error}}_{\mathrm{Forecast}}∼1+\mathrm{Aggregation}+\mathrm{Timebin},$$

where Error_Forecast_ (i.e. Brier score) is the target variable, Aggregation is the aggregation treatment of the data (i.e. a dummy variable representing ‘individual’, ‘1st order compromise’, or ‘2nd order compromise’), and Timebin is the time window relative to question closure (i.e. a dummy variable representing the nine different time intervals analysed). The summary statistics of the GLM are shown in electronic supplementary material, table S1.

The comparisons of the performance curves in figure 1*d* were done in two steps. First, a Kruskal-Wallis test was performed for each time window, in order to test whether the data from the three levels of aggregation originate from the same global distribution. For each of the time windows where the Kruskal-Wallis test *p* < 0.05, we further performed multiple comparisons with Mann-Whitney *U*-tests, and corrected the obtained *p*-values via Bonferroni. The results are shown in electronic supplementary material, table S2.

## 4. Results

To investigate the accuracy of predictions relative to the time left to the event deadline (event horizon, time zero), we calculated Brier scores from the probability forecasts within several overlapping time windows.

We start by examining average individual forecast accuracy and confidence (for definition see Methods; figure 1*b,c*) starting from around 100 days before the IFP deadline. Two general patterns are observed. First, confidence exhibits a late rise for all three aggregation levels, but for individual forecasts the confidence is generally lower than the two compromise aggregation levels. Second, accuracy does not follow a similar pattern. Instead, it shows an incrementally rising trend as question deadline approaches, with a small qualitative dip around the 55 day mark for individual and 1st order compromise aggregation levels. A similar result is observed when we look at the forecasting error (i.e. Brier score, equation (3.1)) across time (figure 1*d*). Given that Brier score combines the accuracy and confidence into a singular measure of error, we see very clearly that both the individual and 1st order compromise forecast errors (figure 1*d*, blue solid and red short-dashed curves) are maximum in the beginning (due to low confidence) and in the 55 day mark, due to the above mentioned dip in absolute forecasting accuracy.

To investigate the presence of a general effect of data treatment (aggregation level) and/or time to deadline on the forecast accuracy, we ran a GLM as described in the methods (equation (3.3)). We found that both variables have a significant effect (*P*_Aggregation_ = 5.88 × 10^−16^, *P*_timebin_ = 1.57 × 10^−26^). The summary statistics of the GLM are shown in electronic supplementary material, table S1.

We then examined our first hypothesis to see whether averaging the forecasts of team members with one another and drawing a compromise forecast would demonstrate the standard wisdom of crowd effect, as exemplified by prediction markets [4]. We averaged the forecasted probabilities across team members to obtain a 1st order compromise forecast, from which we then calculated the forecasting error (figure 1*d*, red short-dash curve). This simple way of arriving at compromise answers, i.e. averaging the probability predictions across participants within a team, shows a significant tendency of lower forecasting error (i.e. Brier score) for every time bin analysed (electronic supplementary material, table S2). The data underlying (i.e. less collapsed) figure 1*d* further shows that there is great variability of Brier scores through time, both across teams and questions (electronic supplementary material, figure S2). This suggests that, although the 55 day mark before deadline seems to indicate a qualitative change in the forecasting accuracy, there is no uniform effect across teams nor questions.

The profile of the individual and 1st order compromise curves is very similar, showing a roughly constant absolute difference of the curves across time. Nevertheless, an absolute difference of decreasing the forecasting error will entail a different ‘amount of improvement’, depending on which section of the Brier score scale we are focusing on. To normalize the effect, we calculated the Modulation Index (MI, equation (3.2); figure 1*e*). In essence, the larger the normalized accuracy of the compromise relative to the individual answers, the larger the MI will be. This way we can see that 1st order compromise forecasts are not only more accurate than individual forecasts, but that the improvement becomes greater as the event horizon approaches.

Having shown that aggregating individual answers to create a compromise forecast improves accuracy, and inspired by previous reports where aggregation of compromise answers improved the accuracy of both questions about the present [27] and about the future [13], we tested if further aggregation would render even more accurate forecasts. For this, we took the 1st order compromise forecasts and averaged them across teams, obtaining a 2nd order compromise forecast (figure 1*d*, yellow long-dash curve). Although statistical testing showed that the 2nd level forecast is also better than the individual aggregation, it did not show a statistically relevant difference between the 1st and 2nd order aggregation performance (electronic supplementary material, table S2). Having said that, we do observe a slight tendency of improved forecasts with the second level of aggregation that, notably, does not show the same bump in forecasting error around two months (55 day mark) prior to the event horizon.

Navajas *et al*. [27] noted that members of statisticized groups (sG, defined as ‘nominal groups that are constructed through statistical reorganization of the data’) have not had any social interaction with each other. As a consequence, if aggregation of opinions within natural groups and sG show a difference, then that difference cannot be purely due to statistical phenomena such as error canceling which are expected to happen in sG. For example, social interaction may increase correlation among opinions in a natural group, reducing the beneficial impact of error cancellation. Alternatively, information flow from informed to uninformed group members may benefit natural groups. As such, the comparison between sG and natural groups can be informative.

The improved forecasting accuracy of 1st level compromise compared to individuals (blue solid and red short-dash curves in figure 1*d*) will not be a consequence of team interaction, if it does not change across all methods of aggregation (i.e. natural interacting teams versus sG). To examine this question in more depth, we tested whether the benefit (i.e. the forecasting improvement) of aggregation remained after shuffling the participants across teams, before calculating the Brier scores (electronic supplementary material, figure S3). Indeed, for every time window, the team compromise (1st order) shows a lower forecasting error than the individual performances also for the shuffled data. Therefore, we conclude that the benefit of compromise is indeed independent of the group deliberation, consistent with what has been shown before by Navajas *et al*. [27] and Dezecache *et al*. [13].

## 5. Discussion

Much has changed since Cresus's attempt to find who could best see into the future. The Good Judgement Project has consistently shown that the more interactive forecasters were also the better ones. Yet, the best forecasters, like ancient oracles, continue to enter the competition individually: their forecasting is informed and discussed with others, but the forecasting error is calculated for each individual. By contrast, we show the benefit of combining individual forecasts in a single team compromise and calculating the error for such collective predictions. Importantly, this benefit holds across time, including at the earlier stages when predictions are arguably more difficult. Introducing a second-order aggregation of compromises, though not significantly better, is even more stable through time.

A residual concern regarding our design and analyses is that it is based on a limited sample (i.e. it included 382 outcomes of events). Thus, like any other statistical analysis, our findings should be treated with caution and care. It is conceivable that there might be some correlation in the 32162 Brier scores that have gone into our model (induced by the fact that the outcomes are exactly the same—either 0 or 1—for any given event) and other researchers may prefer other ways to explicitly account for them than what we have done here. For example, one might argue that some sort of clustering of errors by question (382 of these) or hierarchical model is needed. Similarly, it may be good to do some clustering by team, individual forecaster, and/or time bin as well. All the data (original, and processed) are available online and we encourage our interested readers to try out their own preferred analysis. In good faith, we doubt that the overall findings would change, but other alternative approaches may add to the reliability and robustness of our findings.

Navajas *et al*. [27] also highlighted that collective intelligence could benefit from a ‘divide to conquer’ principle: instead of averaging all the individual answers within a large crowd, it is better to first divide the crowd in small groups, let them discuss and then aggregate their collective answers. Here we do more than extend this solution to forecasting and to considerably more data. We are capable of showing that the benefit holds in a real-life contest and across time. Our method also differs in that it distinctively targets the benefits of aggregating groups' answers, rather than the whole crowds: in Navajas *et al*. [27] the groups were also the only ones to benefit from interactions, while the classic wisdom of crowds was calculated on responses before interaction. In our case, interaction is kept constant across both types of aggregation, i.e. averaging across individuals within a group or across groups. Finally, the consensus used in Navajas *et al*. [27] was achieved for instance through multiple rounds of deliberations, and feedback in the Delphi forecasting method (famously named after the oracle favoured by Cresus, see [32], and [33] for recent experimental examination) is either fragile or time-consuming, because people with different views may not easily accept to converge on a single answer. By contrast, compromise remains robust and easy-to-calculate.

Many forecasting methods have been proposed that allow us to see the future better and sooner, and the comparison needs to consider the effects of context, people and costs (see [34] for review and a similar point). The benefits of our approach however also go beyond accuracy: introducing actual compromise in the forecasting instructions can bring additional benefits to the forecasters themselves, by reducing the eventual stress associated with providing individual predictions. Previous work ([35,36], see also [37]) shows that individuals feel less burdened and stressed when they know that collective, rather than individual responses, are taken into consideration. This stress or responsibility reduction could be particularly effective in a competitive forecasting tournament, when individuals know that their performance through time is tracked.

Several studies have suggested that it is beneficial that forecasters are free to choose which questions to address, and which to skip ([38,39], but see [40]), meaning that they are not forced to express a confidence judgement on issues where they feel they have no clue. Eventually, one could let the forecasters themselves decide whether they want their forecast to be counted alone or combined as a compromise.

Whether collective intelligence can be observed in GJP data or not was not self-evident and has been a question for the practitioners of the field since the day the data was made public. What is intuitive for a collective intelligence expert is not necessarily intuitive for a forecasting expert. Even for experts in Collective Intelligence, Wisdom of Crowds depends critically on independence of opinions, and social interaction among crowd members is expected to increase correlations in opinions and cancel the wisdom that one may expect to achieve from error cancellation.

Following Dezecache *et al*. [13] laboratory results, here we demonstrate the extension of the structured-crowds strategy to another domain using a much larger dataset obtained under a much broader and less controlled set of conditions. Together, our results provide ‘field evidence’ for the wisdom of structured crowds in forecasting. Furthermore, this work helps lay the foundation to apply the ‘easy-to-calculate’ compromise of forecasts to real-world questions and, more importantly, in real-world conditions (i.e. much less controlled than in traditional laboratory settings).

When Cresus had to decide whether to attack the Persian army of Cyrus the Great, he consulted not just one but two oracles. History retains that he misinterpreted the two as encouraging an attack, which led to the loss of his empire. What we show here is that Cresus made at least two other mistakes along the way, as he should not only have let the two oracles discuss in probabilistic terms with each other, but also trust a compromise between their judgements.

## Data accessibility

The original data is available at the Good Judgment Project [29]). All codes to process and analyze the data, alongside the processed data are available for download at: https://gin.g-node.org/dnferreiro/GJP_revisited.

Supplementary material is available online [41].
